# Responses of Phyto- and Zooplankton Communities to *Prymnesium polylepis* (Prymnesiales) Bloom in the Baltic Sea

**DOI:** 10.1371/journal.pone.0112985

**Published:** 2014-11-13

**Authors:** Elena Gorokhova, Susanna Hajdu, Ulf Larsson

**Affiliations:** 1 Department of Ecology, Environment and Plant Sciences, Stockholm University, Stockholm, Sweden; 2 Department of Applied Environmental Science, Stockholm University, Stockholm, Sweden; University of Vigo, Spain

## Abstract

A large bloom of *Prymnesium polylepis* occurred in the Baltic Sea during the winter 2007 – spring 2008. Based on numerous reports of strong allelopathic effects on phytoplankton exerted by *P. polylepis* and its toxicity to grazers, we hypothesized that during this period negative correlations will be observed between *P. polylepis* and (1) main phytoplankton groups contributing to the spring bloom (i.e., diatoms and dinoflagellates), and (2) zooplankton growth and abundance. To test these hypotheses, we analyzed inter-annual variability in phytoplankton and zooplankton dynamics as well as growth indices (RNA∶DNA ratio) in dominant zooplankton in relation to the *Prymnesium* abundance and biomass. Contrary to the hypothesized relationships, no measurable negative responses to *P. polylepis* were observed for either the total phytoplankton stocks or the zooplankton community. The only negative response, possibly associated with *P. polylepis* occurrence, was significantly lower abundance of dinoflagellates both during and after the bloom in 2008. Moreover, contrary to the expected negative effects, there were significantly higher total phytoplankton abundance as well as significantly higher winter abundance and winter-spring RNA∶DNA ratio in dominant zooplankton species in 2008, indicating that *P. polylepis* bloom coincided with favourable feeding conditions for zooplankton. Thus, primary consumers, and consequently also zooplanktivores (e.g., larval fish and mysids), may benefit from haptophyte blooms, particularly in winter, when phytoplankton is scarce.

## Introduction

In 2007–2008, a protracted bloom dominated by *Prymnesium polylepis* (Manton & Parke) Edvardsen, Eikrem & Probert, former name *Chrysochromulina polylepis* [1], occurred in the Baltic Sea [2]. The bloom started in late November – early December 2007, and the abundance increased over time, reaching maximum in March–May 2008 [2]. The bloom raised concerns regarding possible negative effects on phytoplankton competitors, grazers and secondary consumers in the food web. Various Prymnesiales, including *P. polylepis*, being a regular component of marine phytoplankton, are known to produce haemolytic and ichthyotoxic compounds [3], which have been reported to be harmful for a variety of primary and secondary consumers [4]. In field and laboratory studies, *P. polylepis* was found to suppress motility and growth in algae, heterotrophic protists and pelagic bacteria [5]–[8], and to inhibit growth and reproduction or even increase mortality in mesozooplankton grazers [5], [9]. Also, severe toxic effects on benthic communities and farmed fish were observed in areas with high concentrations of *P. polylepis* [10] and in experiments with eggs and larvae of the ascidian *Ciona intestinalis* and the blue mussel *Mytilus edulis* [11].

In May to early June of 1988, an exceptionally large bloom of *P. polylepis* occurred in the Norwegian and Swedish coastal waters of the Skagerrak and the Kattegat [12], [13]. The bloom caused extensive mortalities of various organisms throughout the food web, including bacteria, phytoplankton, zooplankton, benthic macroalgae and fauna, and demersal and pelagic fish [14]. Following the bloom in 1988 and smaller recurrent blooms thereafter, toxic haptophytes were suggested to pose a permanent threat to Scandinavian mariculture and wildlife [13], [14]. Therefore, there was a general expectation that also the *P. polylepis* bloom in the Baltic Sea might cause adverse effects in the ecosystem.

It has also been suggested that toxic haptophytes (including Prymnesiales) may not only cause occasional conspicuous bloom effects, such as fish kills, but also may impact pelagic food webs in coastal waters, by constraining the zooplankton feeding and recruitment even at commonly occurring sub-bloom concentrations [15]. Methodologically, detecting such effects *in situ* is, however, very challenging, because top-down effects on zooplankton abundance obscure bottom-up effects, i.e. mortality due to predation makes it difficult to detect changes due to nutrition. To identify bottom-up effects, measurements of growth or metabolic rates are instrumental; these, however, are laborious to implement in field studies and monitoring. There is an increasing recognition that biochemical indices can provide a suitable alternative and/or complement existing methods, to facilitate assessment of *in situ* growth and physiological condition in zooplankton [16]. To investigate effects of algal blooms (e.g., diatoms and dinoflagellates [17]–[19] and cyanobacteria [20]) on mesozooplankton grazers, RNA-based indices have been applied and found informative. The rationale behind these indices is that cellular RNA allocation (mainly ribosomal RNA) is positively related to the rate of protein synthesis and can be used as a proxy for growth potential. This could be done by assaying the total RNA content either on individual weight basis or normalized to DNA concentration, which is quasi-constant for a given number of cells [16].

In this study, we link phytoplankton and zooplankton dynamics during 2007–2008 and compare these observations to their long-term variability to reveal whether the high abundance of *P. polylepis* coincided with any adverse effects on the standing stocks of co-occurring algae and on growth and population abundance of zooplankton grazers. In zooplankton, we used RNA∶DNA ratio in dominant zooplankters, copepods and rotifers, as a proxy for short-term growth potential.

## Materials and Methods

### Ethics statement

The sampling was conducted within Swedish National Marine Monitoring Programme (SNMMP) and SYVAB's marine monitoring program in Himmerfjärden Bay (Himmerfjärden Eutrophication Study; www2.ecology.su.se) in the Baltic Sea, and no specific permissions were required for the sampling locations of this study. Also, we did not require an ethical approval to conduct this study as no animals considered in any animal welfare regulations and no endangered or protected species were sampled.

### Study areas and data origin

**Figure 1:**
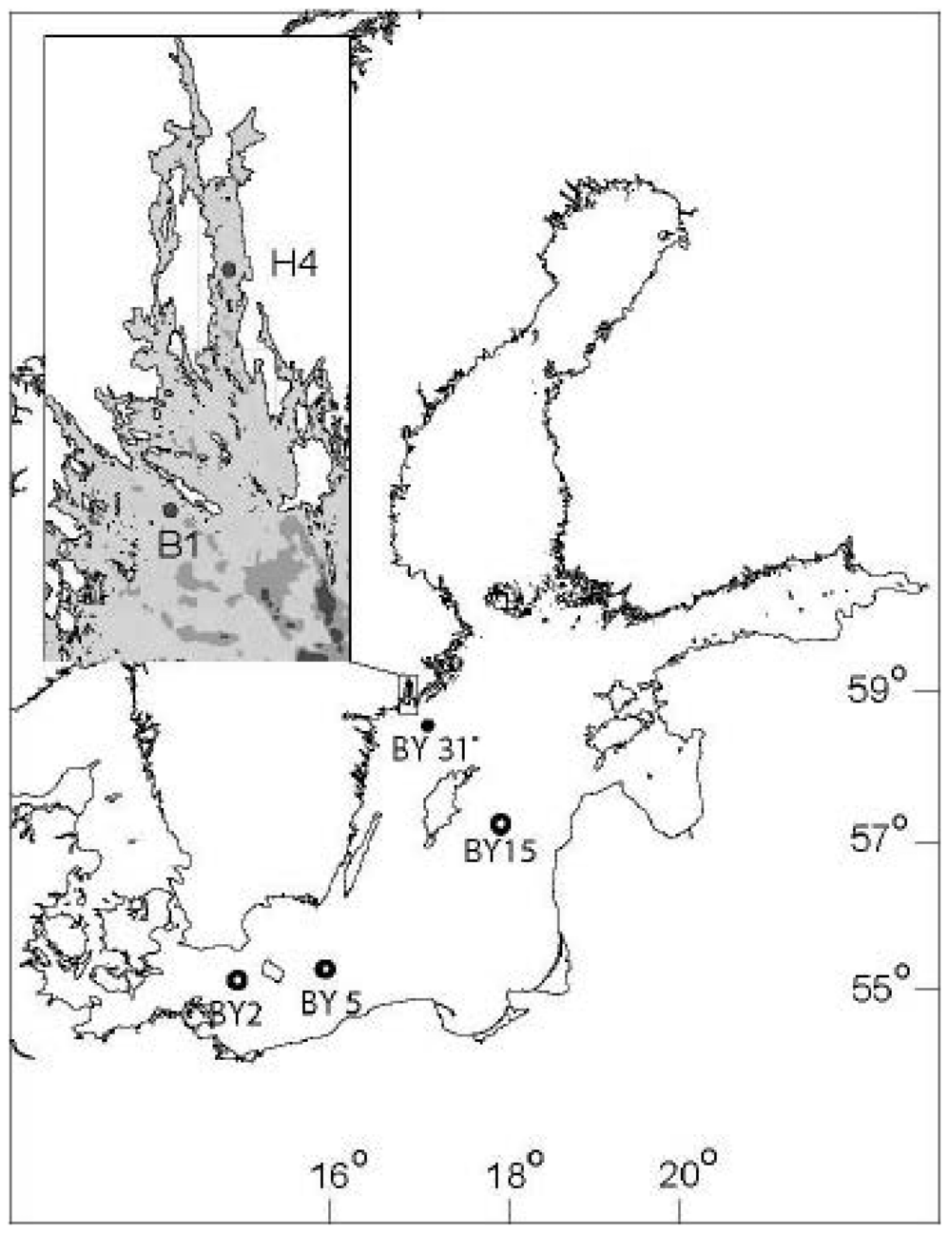
Study area and sampling stations in the central (stn BY15; bottom depth 249 m) and southern basins (stn BY5 and BY2; bottom depth 91 and 48 m, respectively) of the Baltic Sea proper, and in its northern part (stn H4, B1 and BY31; bottom depth 30, 40 and 459 m, respectively). The northern stations represent an inshore-offshore gradient and differ in salinity, available nutrients, phytoplankton development, and production. Station H4 is located in the middle of the Himmerfjärden Bay moderately eutrophied by discharges from a municipal sewage treatment plant. Station B1 is an open coastal station, outside the area influenced by the sewage treatment plant discharge [21], while stn BY31 (Landsort Deep) is the deepest offshore monitoring station in the Baltic Sea.

The Baltic Sea is a non-tidal brackish water estuary comprised of a series of large basins. In the study area (Figure 1), salinity increases southward from 5 to 8, and water temperature varies from near 0°C during winter to 15–20°C during summer. The phytoplankton and zooplankton abundance data used in this study originated from six monitoring stations (Figure 1) sampled in 2007 and 2008 and located in the central (stn BY15) and southern (stns BY5 and BY2) basins of the Baltic Sea proper, and in its northern part (stns H4, B1 and BY31). The central and southern stations were visited once a month, while the northern stations were sampled one to four times a month, depending on the season. The zooplankton RNA∶DNA data were available only for the stations in the northern Baltic proper, where samples are routinely collected and preserved for molecular analyses [22].

### Sampling and sample analysis

At each station, salinity and water temperature were measured by a Conductivity, Temperature, Depth Sensor (CTD) (Meerestechnik Elektronik GmbH) on each sampling occasion. Plankton samples were collected and analyzed according to the Baltic Sea monitoring guidelines [23]. Integrated phytoplankton samples were collected by a plastic hose (inner diameter 19 mm, length 10, 14 or 20 m depending on station) and preserved with acid Lugol's solution.

Phytoplankton (>2 µm) were counted after sedimentation in 10 or 25 mL chambers using an inverted microscope with phase contrast. Microphytoplankton were counted on the half/whole chamber bottom or in diagonals with a 10× objective (total magnification 150×). Nanoplankton were counted in one or two diagonals with a 40× objective (total magnification 600×). In each sample, a minimum of 50 units (cells/colonies/filaments) of the dominating species were enumerated, giving a maximum counting error of ±28% corresponding to a 95% confidence limit for the counts; maximum error for total count per sample was less than ±10%. Biovolumes of phytoplankton cells were calculated using the HELCOM taxa-specific biovolume table http://www.ices.dk/marine-data/Documents/ENV/PEG_BVOL.zip.

The species-level identification of Prymnesiales requires electron microscopic investigation and/or molecular approaches [2]. As these are not an option in routine phytoplankton analysis, we use size based enumeration, which is the recommended practice for Baltic phytoplankton analysis [24]. Here, we focus on two size classes of Prymnesiales: 6–10 µm and >10 µm, which are relevant for the analysis of *P. polylepis* dynamics. We assumed that during the winter-spring 2007–2008 all *Prymnesium* cells >10 µm in the study area were the alternate cell type of *P. polylepis*; this was based on the molecular identification of cells comprising the bloom [2]. Using light microscopy of Lugol-preserved samples, the smaller authentic cell type of *P. polylepis* (6–10 µm) cannot be reliably distinguished from other Prymnesiales of the same size [25]. Therefore, the group Prymnesiales 6–10 µm represents potentially a multispecies group including *P. polylepis* and other Prymnesiales of this size [2], but also other species (e.g., *Haptolina ericina*, *H. hirta*).

Zooplankton samples were taken by vertical tows in the upper 30 m using a 90 µm WP2 net (diameter 57 cm); sampling frequency was the same as for phytoplankton, with a few missing dates, when rough weather precluded net sampling. At stns H4, B1 and BY31, a subsample from each tow was taken for the RNA∶DNA analysis by preserving randomly selected zooplankton with RNA*later* in bulk; these samples were stored for 12 to 24 months at −20°C until the nucleic acid analysis [22]. The rest of the single-tow sample was preserved in 4% borax buffered formaldehyde for species identification and population analysis. In these samples, mesozooplankton were analysed following the standard protocol of the Baltic Sea Monitoring Programme [23]; biomass was calculated using individual species- and stage-specific weights [26]. Replicate subsamples [27] were counted (≥500 specimens) with an inverted microscope (Leitz fluovert FS, Leica) at 80× magnification. Copepods were classified according to species, developmental stage (nauplii, copepodites CI–III, CIV–V, and adults), and sex, whereas cladocerans were classified to species, maturity (females) and sex.

### Zooplankton RNA∶DNA ratio

To enable year-round comparable data for the years 2007 and 2008, RNA and DNA contents were measured in zooplankton species/stages that were present in all RNA*later*-preserved samples; these were copepods *Acartia bifilosa* females, stages CV–CVI, and rotifers *Keratella quadrata* (females without eggs). Individual specimens were picked from the samples and transferred to Eppendorf tubes (3 replicate samples per sampling occasion; copepods: 3 ind. sample^−1^, rotifers: ∼20 ind. sample^−1^). Nucleic acids were quantified using microplate fluorometric high-range RiboGreen (Molecular Probes, Inc. Eugene, OR) assay after extraction with N-laurylsarcosine followed by RNase digestion [28], [29]. Fluorescence measurements were done in triplicate for each sample, standard, and negative control using FLUOstar Optima (filters: 485 nm for excitation and 520 nm for emission) reader and black solid flat-bottom microplates (Greiner Bio-One GmbH). Mean standard curve slope ratio (m_DNA_/m_RNA_) was 1.87.

### Statistics and data analysis

The environmental data (temperature and salinity) as well as phytoplankton and zooplankton data used in all analyses are available from www.smhi.se (Swedish Meteorological and Hydrological Institute; SHARK database) and www2.ecology.su.se/dbhfj/index.htm (Himmerfjärden Eutrophication Study). The time series on zooplankton RNA∶DNA ratio are provided as Supporting Information (Table S1).

To test whether the presence of *Prymnesium polylepis* coincided with decreased stocks in other species, mean monthly values of phyto- and zooplankton stocks were compared between the year 2008 (*P. polylepis* present) and (1) year 2007 (*P. polylepis* absent) by paired t-test (all stations), and (2) the long-term means (1985–2006) of relevant phytoplankton and zooplankton taxa by one-sample t-test (stns B1, H4 and BY31). In these comparisons, two periods based on the *P. polylepis* seasonal development were considered: the entire year (January–December) and the period of the *P. polylepis* occurrence in the water column (January–June). The phytoplankton variables included total phytoplankton biovolume and taxonomic groups that have been reported to respond to the allelopathic effects of *Prymnesium* species in field and laboratory studies, i.e. diatoms, dinoflagellates, cryptophyceans, prasinophyceans and ciliates [5], [30]–[33]. For all analyses, the abundance and biovolume data were Box-Cox transformed to approach normal distribution.

To explore associations between zooplankton growth and standing stocks of main zooplankton groups and dominant species, abundance of *P. polylepis* and Prymnesiales 6–10 µm and environmental factors, such as North Atlantic Oscillation (NAO) index, monthly anomalies of sea surface temperature (SST, 0–10 m) and salinity (anomalies from the long-term data, 1985–2006), were explored using Principal Component Analysis (PCA). The monthly NAO index values were taken from the Climate Prediction Center, Washington, DC (www.nnic.noaa.gov/data/teledoc/nao.html). A high, positive winter NAO index indicates mild and rainy winters, while low, negative values occur during cold winters over Europe. The PCA was performed on standardized data and a similarity matrix based on Pearson product-moment correlation coefficient and using average monthly abundance data for phyto- and zooplankton in 2007 and 2008. To cover the entire gradient in bloom magnitude, two time periods were considered: December 2007 to May 2008, corresponding to the pre-bloom, bloom and early post-bloom of *P. polylepis*, and June 2008 to November 2008, corresponding to the late post-bloom when *P. polylepis* abundances were either very low or none.

To compare zooplankton growth between the years and taking into account seasonal and spatial variability, a generalized linear model (GLM) was applied with zooplankton growth assessed as *RNA∶DNA ratio* as a response variable, and *year* (2007 and 2008), *station* (BY31, B1 and H4), and *season* (winter-spring and summer-autumn) as explanatory variables. Further, GLMs were used to investigate significance of biotic (total phytoplankton biomass, biomass of phytoplankton excluding Prymnesiales, biomasses of Prymnesiales 6–10 µm and *P. polylepis*, and their respective contributions to the total phytoplankton biomass) and abiotic (NAO, SST and salinity) variables for zooplankton RNA∶DNA ratios. To identify the best model (best subset regression) the Akaike Information Criterion (AIC) was used to optimize the number and combination of predictive variables included. When validating the proposed models, the Wald statistic was used to determine the significance of the regression coefficients, a likelihood ratio test was used to evaluate the statistical significance of including each parameter, and model goodness of fit was checked using deviance and Pearson χ^2^ statistics. The change in deviance for single variables was used to estimate the contribution of individual variables to the deviance explained by the final model. Variance inflation factor (VIF) was used as an indicator of multicollinearity; for each independent variable VIF was <3. Residual plots for each model were examined to exclude remaining unattributed structure indicative of a poor model fit. All analyses were conducted using S-plus 8.0 (TIBCO Software Inc.).

## Results

### Prymnesiales dynamics in 2007 and 2008

**Figure 2:**
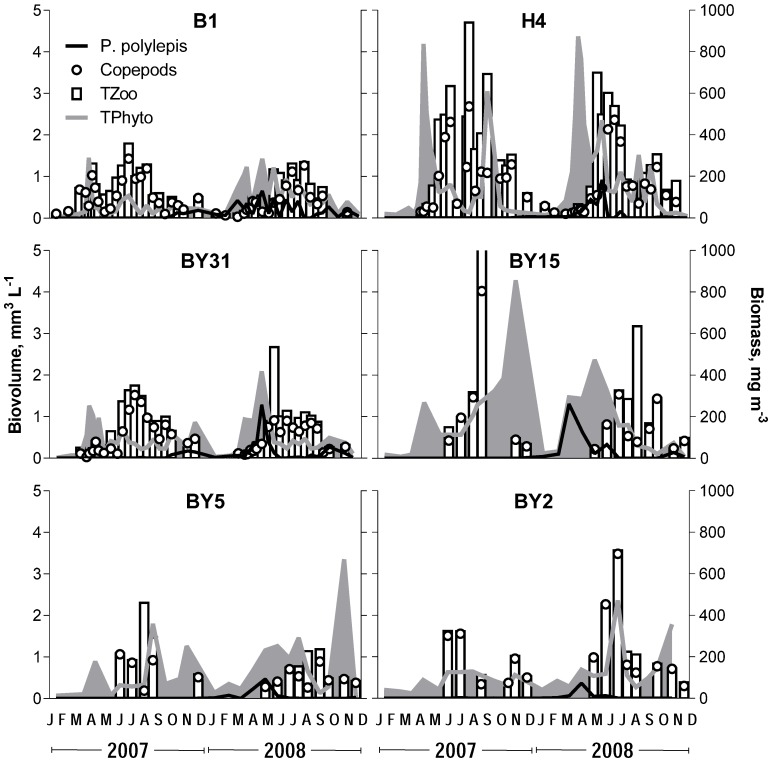
Dynamics of total phytoplankton (TPhyto) and *Prymnesium polylepis* (biovolume, left axis), total zooplankton (TZoo) and copepods (biomass; right axis) during 2007 and 2008 at the monitoring stations in the Baltic proper. Note that in September 2007, TZoo value at stn BY15 is outside the axis limits.

In summer 2007, *P. polylepis* was found at all open sea stations, increasing during autumn and reaching 0.3×10^3^ to 73×10^3^ cells L^−1^ in November–December. At this time, *P. polylepis* contributed up to 70–80% to the total phytoplankton biovolume, with particularly high values at stns BY31 and B1. Moreover, the alternate stage of *P. polylepis*, which has not hitherto been found in phytoplankton samples, dominated Prymnesiales assemblages during winter (Figure S1). In spring 2008, *P. polylepis* became very abundant in the whole Baltic Sea proper (Figure 2). During this time, Prymnesiales 6–10 µm also contributed substantially in all study areas (Figure S1). On an annual basis, *P. polylepis* contributed up to 40% of the total phytoplankton biovolume in the north (stns BY31 and B1), ∼30% at the central basin (stn BY15), and up to −8% in the south (stns BY2 and BY5). At the northern stations, *P. polylepis* occurred during the whole year, except for a short period in summer, when the dinoflagellate *Heterocapsa triquetra* (Ehrenberg) Stein dominated, whereas at the central and southern stations it was found only in low numbers during the second half of the year.

### Phytoplankton stocks and community composition

**Figure 3:**
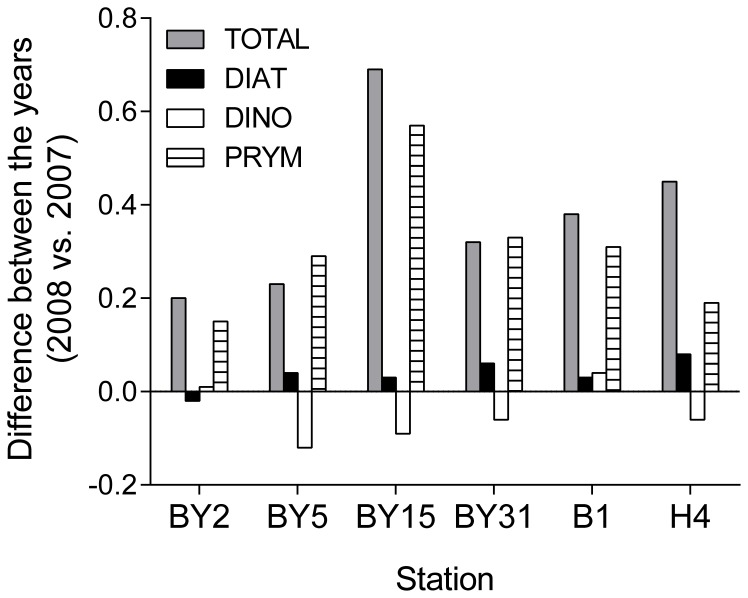
Differences in phytoplankton biovolumes (mm^3^ L^−1^) in January-June between the year 2008 and 2007. Abbreviations: DIAT - Diatoms, DINO - Dinoflagellates, PRYM - Prymnesiales. Stations are ordered south to north.

**Figure 4:**
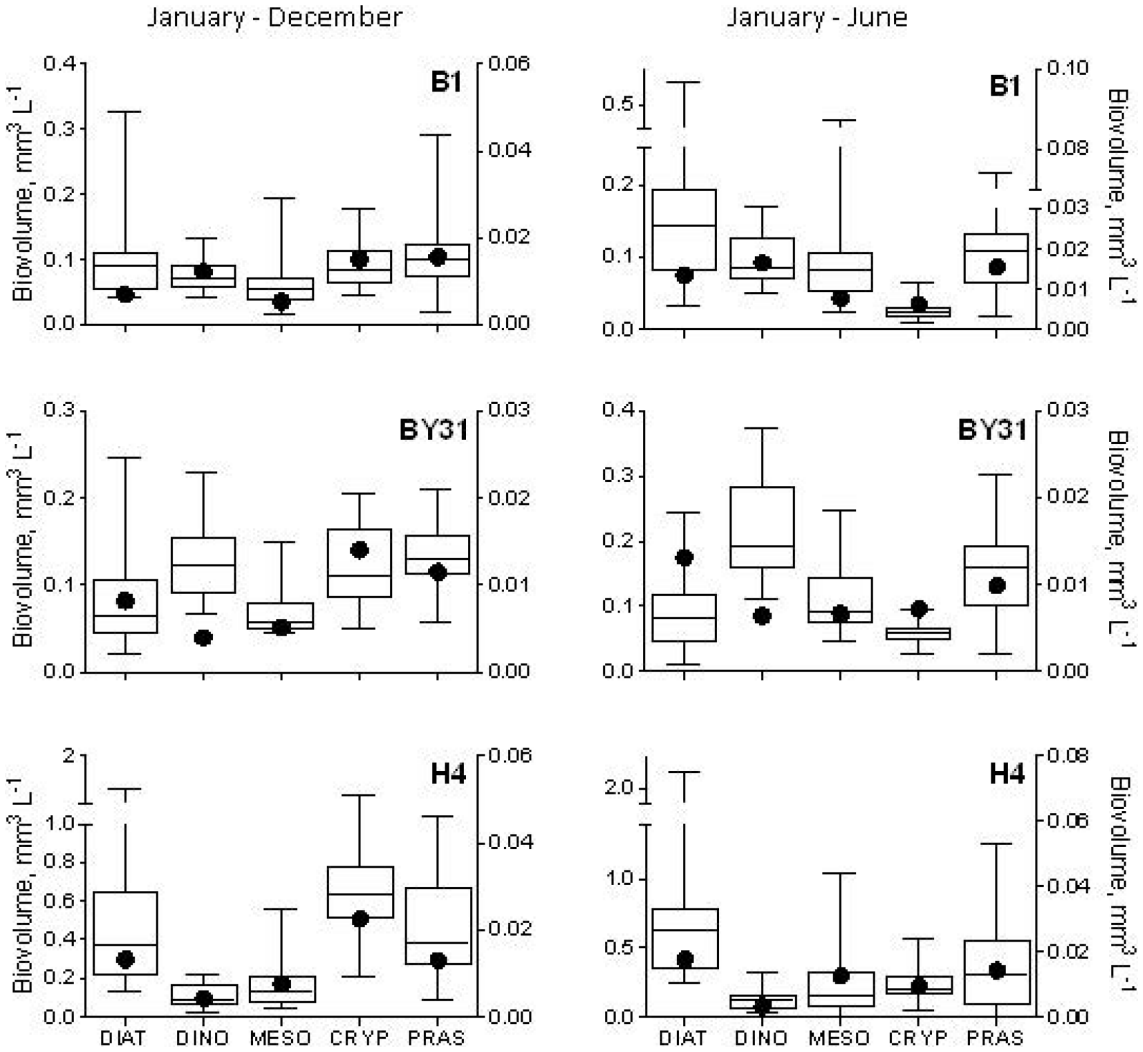
Long-term (1985–2006) variability of group-specific phytoplankton biovolume (mm^3^ L^−1^; box and whiskers with mean, 25–75% range, maximum and minimum) and values observed in the year 2008 (filled circles) at three sampling stations (B1, BY31 and H4) in the northern Baltic Proper. Left panels show yearly datasets (January–December; monthly averages) and right panels show winter-spring data only (January–June). Phytoplankton taxa that have been reported to be affected by *Prymnesium* spp. were included: diatoms (DIAT, left axis), dinoflagellates (DINO, left axis), *Mesodinium rubrum* (MESO, left axis), cryptophyceans (CRYP, right axis), prasinophyceans (PRAS, right axis). Note the different scales on the left and right Y-axes.

For most stations, higher total phytoplankton biovolumes were observed in 2008 than in 2007, with significant differences mainly for the winter-spring period (Table 1; Figure 2). At stn B1, the yearly mean biovolume was more than doubled in 2008 compared to 2007 (0.50 vs. 0.22 mm^3^ L^−1^); moreover, ∼30% difference remained when *P. polylepis* was excluded (0.30 vs. 0.19 mm^3^ L^−1^). In winter-spring, diatoms at all stations, except stn BY2, had higher biovolume in 2008 than in 2007, with overall significant difference between the years (t_5_ = 5.49, p<0.003), while the opposite was observed for dinoflagellates that were generally lower (t_5_ = 3.91, p<0.02; Figure 3). The diatom response was associated with a general increase in *Thalassiosira* species observed at all stations and, to some extent, *Skeletonema costatum* that was higher in the central Baltic proper and the Bornholm basin but lower in the northern Baltic proper (Figure S2). Another algae that were consistently more abundant in 2008 than in 2007 were the euglenoid flagellates *Eutreptiella* spp. and some dinoflagellates from the order Peridiniales (Figure S2). However, other species of this order, such as the chain-forming, spring dinoflagellate *Peridiniella catenata*, showed consistently lower values in 2008 (Figure S2), contributing strongly to the observed decrease in total dinoflagellate stocks during the Prymnesium bloom. When values from the year 2008 were compared to the long-term means for the stations in the northern Baltic proper, the group-specific biovolumes were mostly within the 25–75% range of the long-term variability (Figure 4), except dinoflagellates at stn BY31, where the 2008 values were significantly lower than the long-term mean, both on the yearly basis (t_16_ = 7.99, p<0.0001) and for the January-June period (t_16_ = 7.27, p<0.0001).

**Table 1:** Differences in mean values of total phytoplankton biovolume between the years (2008 vs. 2007) evaluated by Wilcoxon signed rank test using mean monthly values for the entire year (Jan–Dec) and winter-spring (Jan–Jun); p-values: significant effects are in bold, marginally significant in Italics.

Period included	H4	B1	BY31	BY15	BY5	BY2
Jan–Dec	0.151	**0.002**	0.365	0.426	0.164	**0.020**
Jan–Jun	**0.031**	**0.016**	0.467	**0.031**	*0.063*	*0.063*

See also Figure 2.

### Zooplankton communities

For all stations, zooplankton dynamics was similar between the years (Figure 2), with copepods contributing 69–92% and 65–93% to the total zooplankton biomass in 2007 and 2008, respectively. In all areas, the most dominant copepods were *Acartia* spp. (*A. bifilosa*, *A. longiremis* and *A. tonsa*), contributing 45–51% to the total copepod biomass. There were no significant differences in the mean monthly total zooplankton biomass between the years for any of the stations and any of the periods (Wilcoxon signed rank test; p>0.05 in all cases). In December 2007, however, *Acartia* biomass at the coastal stations of the northern Baltic proper was significantly higher than its long-term (1985–2006) mean (p<0.008 in both cases); this translated into significantly higher total zooplankton biomass in the area during this month (p<0.008 and p<0.05 for stns B1 and H4, respectively). Other copepods occurring at higher abundances in 2008 compared to 2007 were *Temora stylifera* and *Centropages hamatus* (Figure S3).

### Zooplankton RNA∶DNA ratios

**Figure 5:**
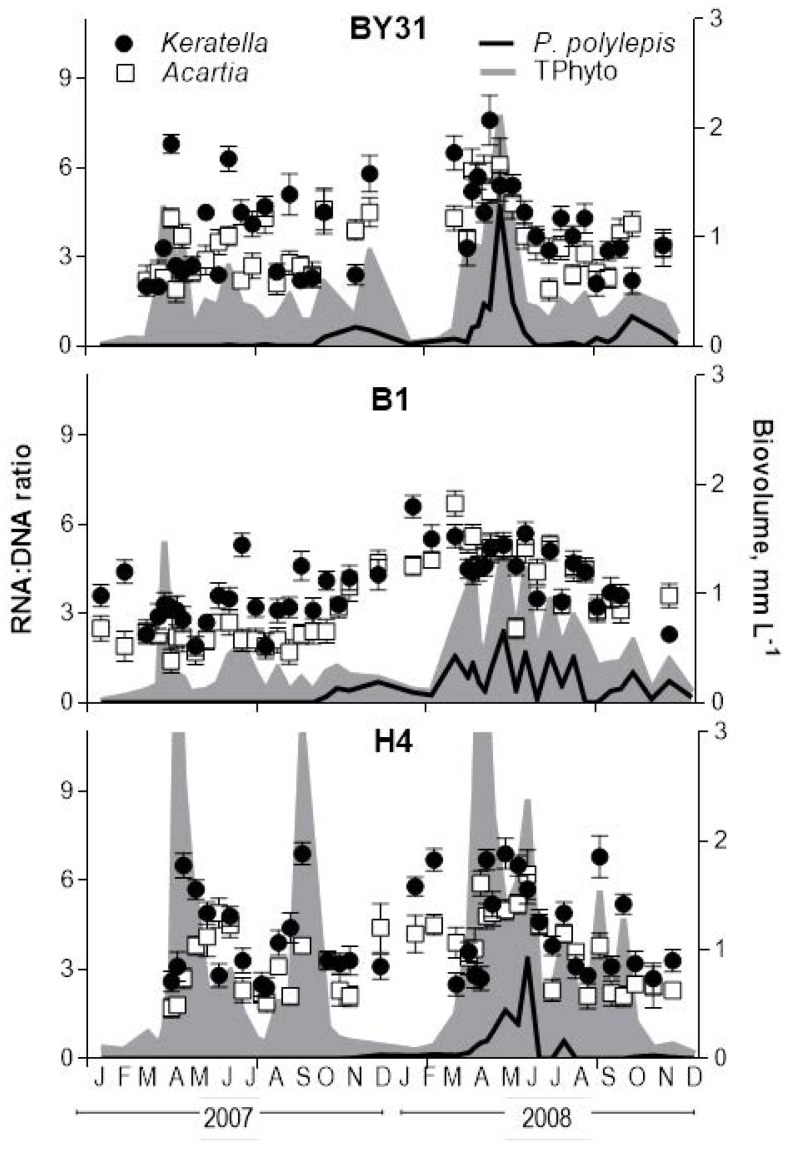
Dynamics of RNA∶DNA ratio (monthly mean ± SD, *n* varies from 5 to 12) in the copepods *Acartia* spp. and rotifers *Keratella quadrata* (left axis) in relation to the total phytoplankton (TPhyto) and *Prymnesium polylepis* (biovolume, right axis) during 2007 and 2008; stn BY31, B1 and H4 in the northern Baltic proper. Note that main peaks for TPhyto at stn H4 are outside the axis limits.

During the study period, RNA∶DNA ratios ranged from 1.4 (stn B1, 2007) to 6.7 (stn B1, 2008) in *Acartia* spp., and from 1.9 (stn B1, 2007) to 7.6 (stn BY31, 2008) in *Keratella quadrata*, generally increasing with increased phytoplankton stocks (Figure 5). For each taxa-specific GLM with *RNA∶DNA ratio* as a response variable and *year*, *station*, and *season* as explanatory variables, a significant *season*×*year* interaction effect was observed (*Acartia*: Wald stat = 10.81, p<0.001; *Keratella*: Wald stat = 5.79, p<0.016). Hence, it was followed up by the season-specific models to test effects of station and year. In winter-spring, RNA∶DNA ratios in both species were significantly higher in 2008 compared to 2007 (*Acartia*: Wald stat = 41.18, p<0.0001; *Keratella*: Wald stat = 13.97, p<0.001) and the effect was consistent for all stations. In summer-autumn, there was a significant *station*×*year* interaction effect for RNA∶DNA ratio in *Acartia* (F = 3.86, p<0.026), with significantly higher ratios in 2008 than in 2007 observed at stn B1 (Wald stat = 20.63, p<0.0001) but not at the other stations (p>0.05 in both cases). No significant effects were observed for RNA∶DNA ratios in *Keratella* for the summer-autumn period (p>0.4 in all cases).

### Effects of Prymnesiales abundance on other plankton groups

**Figure 6:**
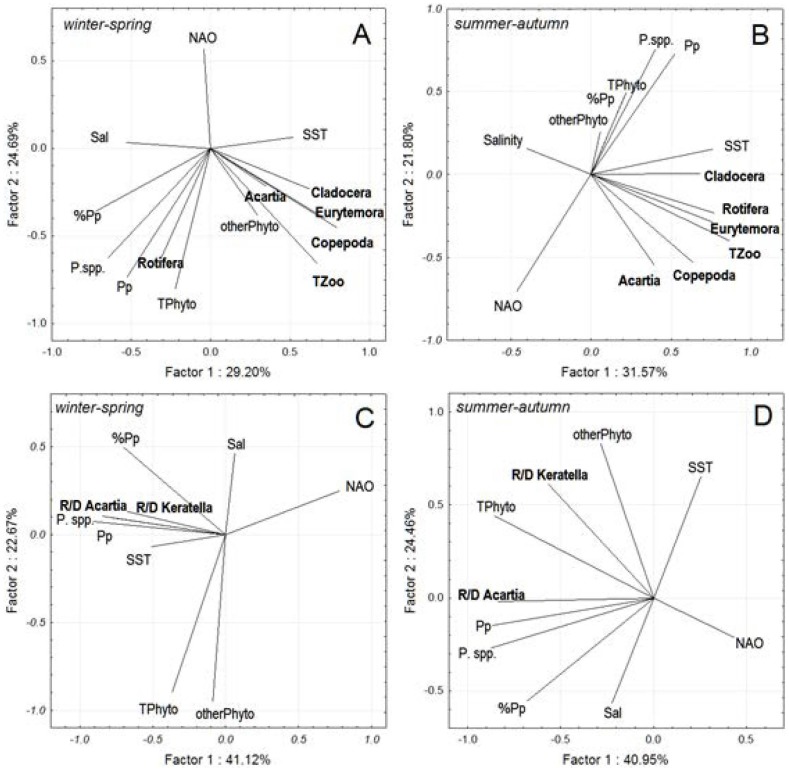
PCA correlation biplot of the two dominant components defined by abiotic (monthly NAO, SST, and salinity [Sal]) and biotic (total phytoplankton biomass [TPhyto], biomass of *Prymnesiales* [P.spp.], phytoplankton biomass excluding *Prymnesiales* [otherPhyto], biomass of *P. polylepis* [Pp], and contribution of *P. polylepis* to total phytoplankton biomass [% Pp]) environmental variables in winter-spring (panels A and C) and summer-autumn (panels B and D) in 2007 and 2008. Panels A and B: Zooplankton biomass (total zooplankton [TZoo], copepods [Copepoda], cladocerans [Cladocera], rotifers [Rotifera], *Acartia* spp. [Acartia], and *Eurytemora affinis* [Eurytemora]) are projected as supplementary variables; data from all sampling stations are used for the analysis. Panels C and D: RNA∶DNA ratios in *Acartia* spp. [R/D Acartia] and *Keratella quadrata* [R/D Keratella] are projected as supplementary variables; data are for stns B1, BY31, and H4.

The correlations between abundance of some zooplankton groups (e.g., *Acartia* spp. and rotifers) and phytoplankton stocks revealed by PCA analysis were more pronounced in the winter-spring period (Figure 6A) compared to the summer-autumn period (Figure 6B), probably owing to greater amplitude of variation in both grazers and phytoplankton during spring. On the correlation biplot defined by the environmental variables (Figure 6), zooplankton abundance was largely unrelated to either the standing stocks of Prymnesiales species, including *P. polylepis*, or their contribution to the total phytoplankton biomass. By contrast, when zooplankton growth indices were considered, the positive associations between RNA∶DNA ratios in dominant zooplankters (*Acartia* spp. and *Keratella quadrata*) and biomass of both small-sized Prymnesiales (6–10 µm) and *P. polylepis* were strong during the winter-spring season (Figure 6C). Moreover, in *Acartia*, these correlations remained during the summer-autumn (Figure 6D).

Existence of the positive relationships between Prymnesiales and zooplankton growth were further supported by GLMs (Table 2). In winter-spring, zooplankton RNA∶DNA ratios responded positively to the increased standing stocks and contribution of *P. polylepis* to the total phytoplankton. In summer-autumn, positive effects on *Acartia* RNA∶DNA ratios were attributed to biomass of small-sized Prymnesiales (6–10 µm) and their contribution to the total phytoplankton, whereas non-Prymnesiales phytoplankton was the main determinant for the rotifer RNA∶DNA ratios. None of the abiotic factors were significant in the *Acartia* models, whereas monthly NAO was a significantly negative predictor in both *Keratella* models.

**Table 2:** Generalized linear model for zooplankton growth indices as a function of abiotic (monthly NAO, SST, and salinity) and biotic (total phytoplankton biomass [TPhyto], biomass and contribution of Prymnesiales [P and %P, respectively], biomass and contribution of *P. polylepis* to TPhyto [Pp and %Pp, respectively] and phytoplankton biomass excluding *Prymnesiales* [otherPhyto]) variables in winter-spring (Dec–May) and summer-autumn (Jun–Nov) during 2007–2008; see Methods for details on data origin and time coverage.

Effect	Estimate	Wald statistic	*p*
I. RNA∶DNA in *Acartia* spp.			
(a) winter-spring			
otherPhyto	0.108	9.108	**0.003**
Pp	0.307	7.138	**0.008**
%Pp	0.008	28.852	**<0.000**
(b) summer-autumn			
otherPhyto	0.194	13.852	**0.000**
P	0.703	26.311	**<0.000**
%P	0.002	1.948	0.123
II. RNA∶DNA in *Keratella quadrata*			
(a) winter-spring			
NAO	−0.074	4.035	**0.045**
%Pp	0.007	26.314	**<0.000**
(b) summer-autumn			
otherPhyto	0.294	27.060	**<0.000**
NAO	−0.005	7.405	**0.007**

Significant effects are in bold face.

## Discussion

Species of the order Prymnesiales, especially *Prymnesium polylepis*, are known to produce harmful blooms [12], [34], [35], with severe impacts on plankton [5], although the toxic effects of these blooms may depend on various abiotic factors [32]. The bloom of the alternate type of *P. polylepis* that occurred in December 2007–May 2008 with maximal observed densities of ∼4×10^6^ cells L^−1^ [2] was relatively small compared to other described blooms of this species, particularly, the extensive toxic bloom in 1988 in the Kattegat-Skagerrak area, where the densities exceeded 70×10^6^ cells L^−1^ [5], [10]. Nevertheless, the Baltic Sea bloom could have induced adverse food web effects, because toxicity and population density in *P. polylepis* seem to be uncoupled [30], similar to the closely related *P. parvum* [36]. Supporting these expectations, reproduction problems were recently reported in breeding eiders, i.e. higher consumers of the Baltic food web, and linked to the bloom [37]. In the latter study, spatial distribution of *P. polylepis* in 2008 closely matched the observed distribution of non-breeding female eiders. The authors suggested that through either toxic or non-toxic pathways, feeding on *P. polylepis* affected local populations of blue mussels, the main benthic food of eiders, and, subsequently, the body condition of adult female eiders and their breeding propensity. It was also suggested that breeding eiders can be significantly affected by species interactions at low trophic levels (i.e., plankton) caused by the bloom; however, our results do not support these speculations.

Contrary to the hypothesized overall negative effects of *P. polylepis* on phytoplankton assemblages, the yearly means of total phytoplankton biomass were significantly higher in 2008 than in 2007 (even when *P. polylepis* was excluded); this holds true both in the south and in the north. Spring stocks of the total phytoplankton, Prymnesiales and diatoms were significantly higher in 2008 than in 2007, while those of dinoflagellates were generally lower (Table 1; Figure 3). Moreover, they were significantly lower than their long-term mean in the open sea area of the northern Baltic proper (Landsort Deep; Figure 4). In the Baltic Sea, spring peak of diatoms is usually followed by that of dinoflagellates [38], and, apparently, in 2008 the Prymnesiales populations successfully competed with the dinoflagellates, resulting in a lower biomass of the latter. Many Prymnesiales are mixotrophs, feeding on bacteria and small algae when nutrients and light levels are low, and, thus effectively compete with other algae [33], [39], [40]. The balance between photosynthesis and phagotrophy appears to be influenced by environmental factors [33], [41]. Indeed, in the northern Baltic Sea proper, biomass of *P. polylepis* was high already in winter, when light availability was extremely low, suggesting phagotrophic nutrition of the dominating alternate type of *P. polylepis*. Moreover, a closely related species, *P. parvum*, has been reported to directly utilize dissolved organic matter (osmotrophy) [42]. The ability to switch between the autotrophic, heterotrophic and osmotrophic nutrient acquisition modes might be one of the reasons why *P. polylepis* successfully maintained population during the winter months reaching high abundances (>10^6^ cells L^−1^) in late spring in the stratified, nutrient depleted conditions. The reported negative effects of dissolved inorganic nitrogen on Prymnesiales [43] support the probable independence of the nitrogen availability and the notion that the latter may have positive effects on competition with autotrophs. Regardless of the ecological mechanisms, our findings suggest that environmental conditions prevailing during the study period were favourable for phytoplankton development, especially in spring 2008, and increase in primary producers cascaded up to higher trophic levels stimulating secondary production.

No negative impact of the *P. polylepis* bloom on planktonic grazers was observed during the bloom, and no carry-over effects were apparent later in the season. On the contrary, higher abundance of most zooplankton taxa (Figure S3) and greater RNA∶DNA ratio of the dominant zooplankton species were observed during winter-spring, indicating favourable feeding conditions for mesozooplankton exposed to the *P. polylepis* bloom in the Baltic Sea. Significantly higher *Acartia* spp. abundance in December 2007 and positive correlations between zooplankton (copepods and rotifers) RNA∶DNA ratio and both Prymnesiale*s* 6–10 µm and *P. polylepis* (Figure 6CD; Table 2) suggest that individual growth in these zooplankters was positively related to both absolute and relative abundances of these algae, i.e. positive bottom-up effects. In summer-autumn, the entire Prymnesiales group appear to correlate positively with the copepods, which may again indicate positive bottom-up effects or, alternatively, similarity in environmental conditions promoting development of Prymnesiales and the copepod growth conditions. By contrast, in rotifers, non-Prymnesiales phytoplankton and NAO were influential on RNA∶DNA ratio during this period. Interestingly, the positive correlations with growth translated into the increased *Acartia* population stocks only in December 2007 (Figure 6AB), which was most probably due to a weaker control of the copepod abundance by fish predation in winter. Thus, regardless of the mechanisms involved, the bloom-promoting conditions were favourable for growth and metabolism of dominant zooplankton grazers in the Baltic Sea.

In addition to the increased food availability promoting growth, mixotrophy of *P. polylepis* may have enhanced trophic transfer efficiency from microbial component of the plankton to mesozooplankton, thereby favoring zooplankton growth. This speculation is based on the observed positive effects of *P. polylepis* isolated in 1988 from the Kattegat on copepod reproduction that were attributed to enhanced trophic transfer efficiency by channeling pico- and bacterioplankton production to the copepods by mixotrophy [44]. Also, the osmotrophic nutrient uptake would support Prymnesiales growth, particularly in low winter light, and provide nutrition to zooplankton. In oligotrophic marine environments, a relatively high bacterivory during winter has been found in haptophytes despite low bacterial abundance [45]. Therefore, although bacteria and picoplankton production are low during this part of year in the Baltic Sea [46], the dissolved organic nitrogen is abundant [47], therefore, the strongest positive effects on *Acartia* spp. observed in winter, could be explained by the unusually high food availability due to Prymnesiales, when mixotrophic and osmotrophic nutrition modes in this group were likely to be substantial.

Studying effects of the Kattegat bloom in 1988, Nielsen et al. [5] observed no inhibition of feeding and growth when a mixture of cultured authentic *P. polylepis* (∼7 µm) and *Rhodomonas* spp. was offered to *Acartia tonsa*, whereas feeding and egg production were negatively affected by incubation in pure cultures of the authentic *P. polylepis*. Moreover, when incubated with monospecific bloom assemblage of the authentic *P. polylepis*, a significantly reduced swimming and egg production together with increased mortality were observed in copepods. In our study, the lack of adverse effects on zooplankton may at least partially be explained by the fact that this study system was more relevant to the mixed algae conditions, where grazers were exposed to natural phytoplankton assemblages containing *P. polylepis*. This is not unusual as after the toxic bloom in 1988, several *P. polylepis* blooms without any harmful effects have been reported in the same area [35], [48].

Toxin production in Prymnesiales is complex and affected by a variety of factors [32]. Therefore, the toxic effects are difficult to compare to other field and experimental studies, because toxicity is not present permanently but is altered through the life cycle in concert with changes in cell morphology, nutrient limitation, salinity, etc. [49], [50]. Moreover, it is associated with growth response to light and temperature [51]. Phosphorus limitation has been implicated in stimulating production and/or release of toxin(s) in *P. polylepis* and associated adverse effects on aquatic life [30]. Notably, phosphorus availability during the *P. polylepis* bloom in the Baltic was above the long-term average (see monitoring data for the relevant stations at: http://www2.ecology.su.se/dbhfj/index.htm), which may explain the lack of negative effects on plankton organisms. Low salinity has also been suggested to reduce the toxic effect [52] and, indeed, *P. polylepis* exhibited low toxicity for the dinoflagellate *Heterocapsa triquetra* at salinities of 5 and 10, typical for the Baltic proper [53], whereas it was highly toxic at a salinity of 29 [5]. Similarly, no fish kills were observed in a salinity of 10 but at 16 and 22 [54]. Finally, the alternate cells of *P. polylepis*, which dominated until May 2008 are less toxic than the authentic type [55]. Therefore, the low salinity in the Baltic Sea could be one of the reasons why no negative effects on grazers and fish were observed during the bloom despite the overall dominance of *P. polylepis*.

All in all, we found no support for the suggested impact on coastal pelagic food webs [37] and constraining zooplankton feeding and recruitment by *P. polylepis* at commonly occurring sub-bloom concentrations [15]. On the contrary, given the observed stimulating effects of bloom conditions and Prymnesiales abundance on zooplankton growth during winter 2007 – spring 2008, and experimental studies demonstrating increased food web efficiency mediated by mixotrophic Prymnesiales species, presence of these algae is likely to enhance feeding conditions for zooplankton, and, consequently, for zooplanktivores (e.g., larval fish and mysids), particularly in winter, when phytoplankton is scarce and toxin production is low.

## Supporting Information

Figure S1 **Dynamics of Prymnesiales assemblages and contribution of** ***Prymnesium polylepis*** **(alternate stage), Prymnesiales 6–10 µm and other Prymnesiales species to the bloom in 2007–2008 at stns B1, H4, BY31, BY15, BY5 and BY2 in the Baltic proper.**
(PDF)

Figure S2 **Phytoplankton taxa showing strong deviations between the study years (2007 vs. 2008) at stns B1 (A), BY31 (B), BY15 (C), BY5 (D), and BY2 (E) in the Baltic proper.** Bars show the difference for the winter-spring period (January–June) between year 2008 and year 2007; numbers over the bars at the highest and lowest range indicate values that lie outside the scale on the Y-axis.
(PDF)

Figure S3 **Zooplankton taxa showing strong deviations between the study years (2007 vs. 2008) at stns B1 (A), BY31 (B), BY15 (C), BY5 (D), and BY2 (E) in the Baltic proper.** Bars show the difference for the entire year (January–December) between year 2008 and year 2007; numbers over the bars at the highest and lowest range indicate values that lie outside the scale on the Y-axis.
(PDF)

Table S1 **RNA∶DNA ratio in the copepod** ***Acartia bifilosa*** **and the rotifer** ***Keratella quadrata*** **at stns BY31, B1 and H4 in the northern Baltic proper (mean monthly values; period 2007–2008).**
(XLSX)
