# Supplementary material for: Responses of Phyto- and Zooplankton Communities to Prymnesium polylepis (Prymnesiales) Bloom in the Baltic Sea
Source: PLoS One. 2014 Nov 13;9(11):e112985. doi: 10.1371/journal.pone.0112985 (PMC4231118; doi:10.1371/journal.pone.0112985)

Responses of phyto- and zooplankton communities to Prymnesium polylepis (Prymnesiales) bloom in the Baltic Sea

Elena Gorokhova, Susanna Hajdu and Ulf Larsson

Figure S1. Dynamics of Prymnesiales assemblages and contribution of *Prymnesium polylepis* (alternate stage), Prymnesiales 6-10 µm and Prymnesiales < 6 µm to the bloom in 2007-2008 at stns B1, H4, BY31, BY15, BY5 and BY2 in the Baltic proper. Note the differences in Y-axis scale among the panels. Authentic stage of *P. polylepis* is included in the 6-10 µm size fraction.

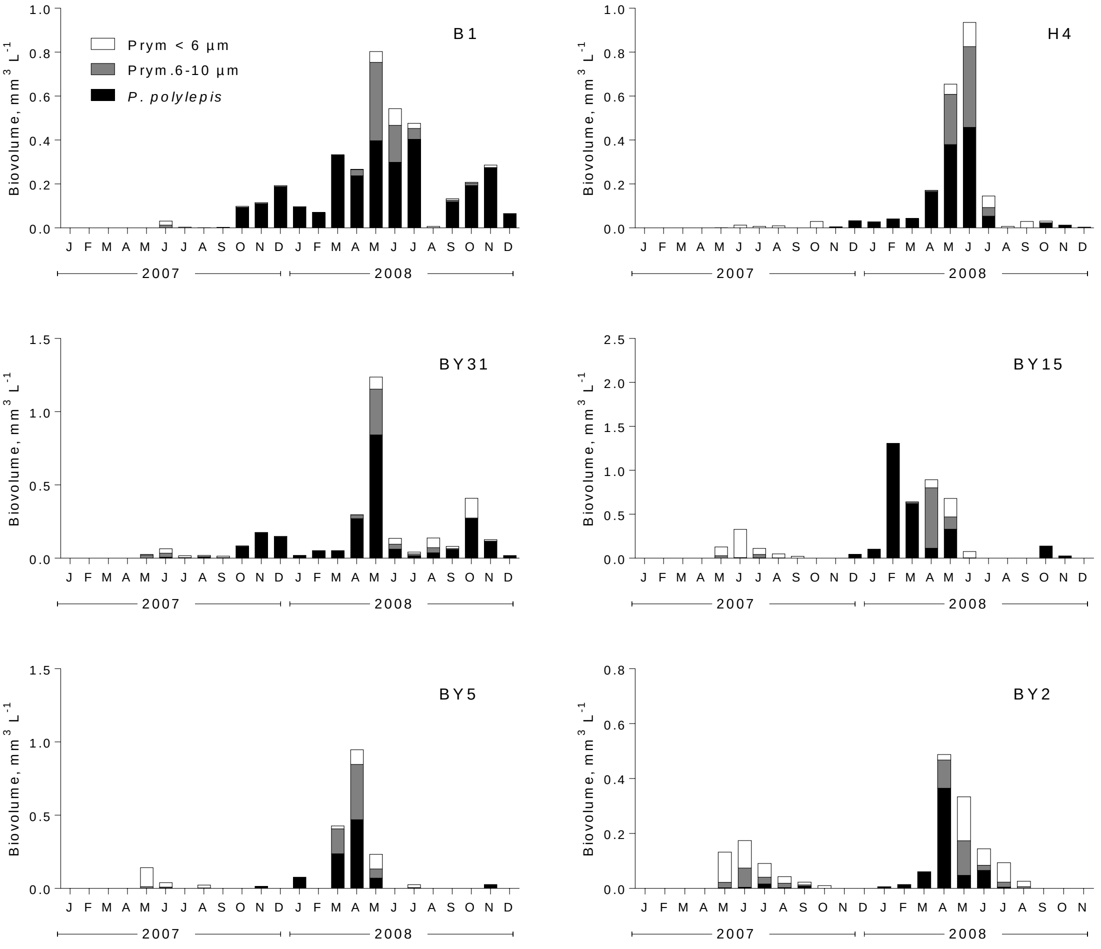

Supplement: Figure S1 — Dynamics of Prymnesiales assemblages and contribution of Prymnesium polylepis (alternate stage), Prymnesiales 6–10 µm and other Prymnesiales species to the bloom in 2007–2008 at stns B1, H4, BY31, BY15, BY5 and BY2 in the Baltic proper. (PDF) [file pone.0112985.s001.pdf]
