# Supplementary material for: Responses of Phyto- and Zooplankton Communities to Prymnesium polylepis (Prymnesiales) Bloom in the Baltic Sea
Source: PLoS One. 2014 Nov 13;9(11):e112985. doi: 10.1371/journal.pone.0112985 (PMC4231118; doi:10.1371/journal.pone.0112985)
Supplement: Figure S2 — Phytoplankton taxa showing strong deviations between the study years (2007 vs. 2008) at stns B1 (A), BY31 (B), BY15 (C), BY5 (D), and BY2 (E) in the Baltic proper. Bars show the difference for the winter-spring period (January–June) between year 2008 and year 2007; numbers over the bars at the highest and lowest range indicate values that lie outside the scale on the Y-axis. (PDF) [file pone.0112985.s002.pdf]

PLoS One │ Supporting Information

Responses of phyto- and zooplankton communities to Prymnesium polylepis (Prymnesiales) bloom in the Baltic Sea

Elena Gorokhova, Susanna Hajdu and Ulf Larsson

Figure S2. Phytoplankton taxa showing strong deviations between the study years (2007 vs. 2008) at stns B1 (A), BY31 (B), BY15 (C), BY5 (D), and BY2 (E) in the Baltic proper.

Bars show the difference for the winter-spring period (January-June) between year 2008 and year 2007; numbers over the bars at the highest and lowest range indicate values that lie outside the scale on the Y-axis. Prymnesiales that showed the highest positive deviation due to the bloom were not included (but see Figure S1 for comparison of Prymnesiales biovolume between years). Abbreviations: Wolo/Scrip/Gymn – Woloszynskia/Scrippsiella/Gymnodinium complex. Stations are ordered north to south.

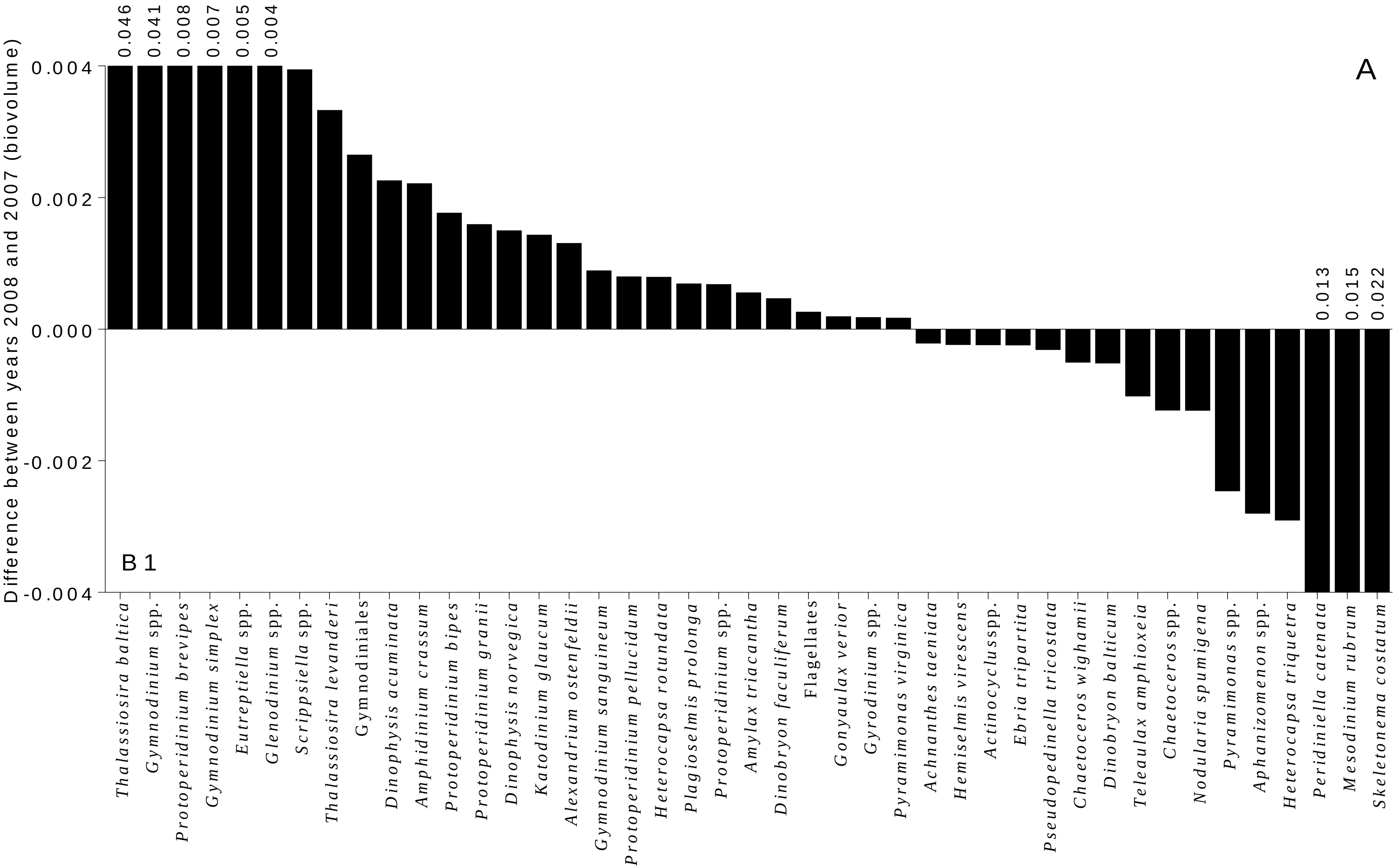

A
B 1
Difference between years 2008 and 2007 (biovolume)
0.004
0.002
0.000
-0.002
-0.004
0.046
0.041
0.008
0.007
0.005
0.004
0.013
0.015
0.022
Thalassiosira baltica
Gymnodinium spp.
Protoperidinium brevipes
Gymnodinium simplex
Eutreptiella spp.
Glenodinium spp.
Scrippsiella spp.
Thalassiosira levanderi
Gymnodiniales
Dinophysis acuminata
Amphidinium crassum
Protoperidinium bipes
Protoperidinium granii
Dinophysis norvegica
Katodinium glaucum
Alexandrium ostenfeldii
Gymnodinium sanguineum
Protoperidinium pellucidum
Heterocapsa rotundata
Plagioselmis prolonga
Protoperidinium spp.
Amylax triacantha
Dinobryon faculiferum
Flagellates
Gonyaulax verior
Gyrodinium spp.
Pyramimonas virginica
Achnanthes taeniata
Hemiselmis virescens
Actinocyclusspp.
Ebria tripartita
Pseudopedinella tricostata
Chaetoceros wighamii
Dinobryon balticum
Teleaulax amphioxeia
Chaetoceros spp.
Nodularia spumigena
Pyramimonas spp.
Aphanizomenon spp.
Heterocapsa triquetra
Peridiniella catenata
Mesodinium rubrum
Skeletonema costatum

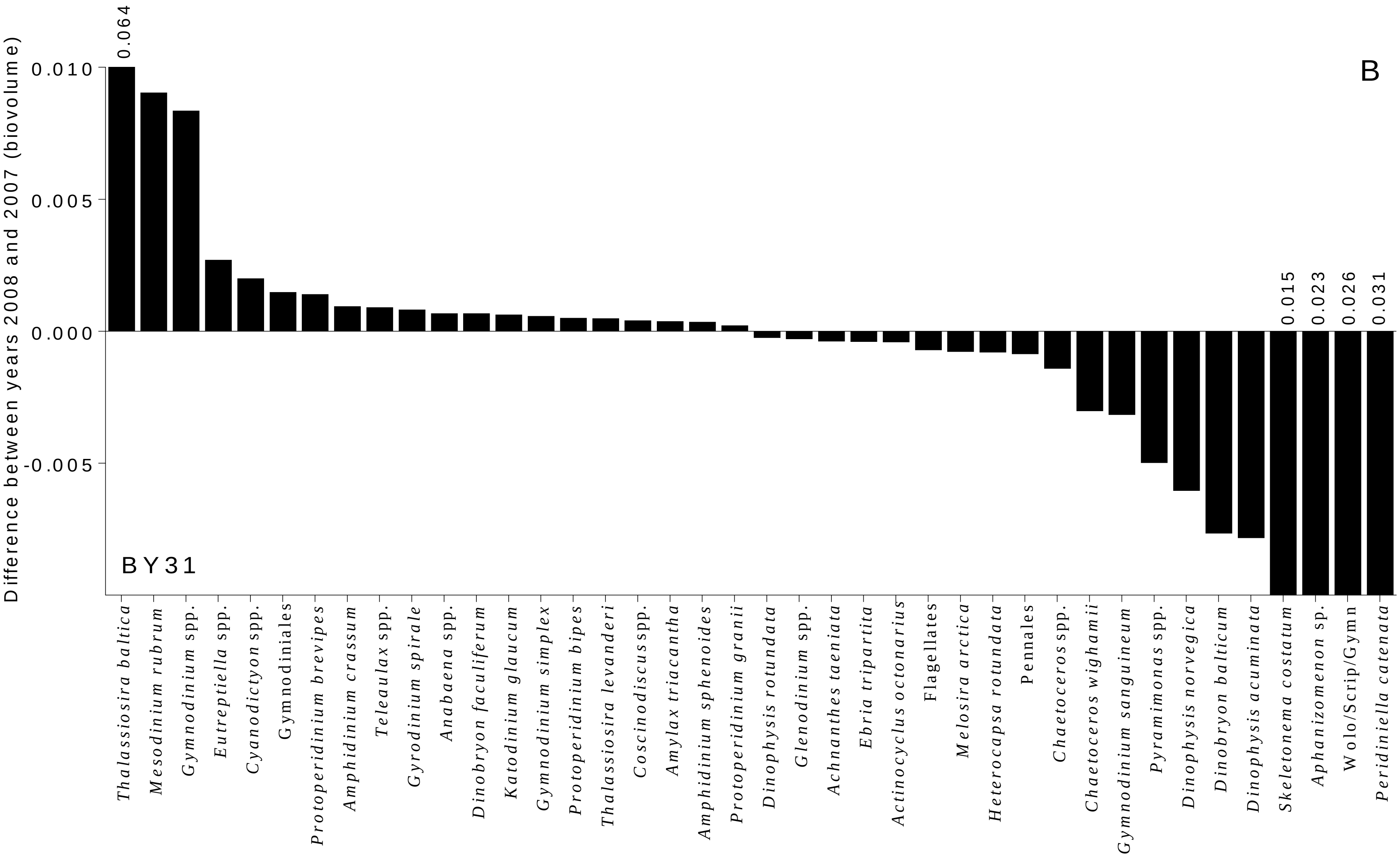

Difference between years 2008 and 2007 (biovolume)
0.010
0.005
0.000
-0.005
0.064
0.015
0.023
0.026
0.031
B
BY31
Thalassiosira baltica
Mesodinium rubrum
Gymnodinium spp.
Eutreptiella spp.
Cyanodictyon spp.
Gymnodiniales
Protoperidinium brevipes
Amphidinium crassum
Teleaulax spp.
Gyrodinium spirale
Anabaena spp.
Dinobryon faculiferum
Katodinium glaucum
Gymnodinium simplex
Protoperidinium bipes
Thalassiosira levanderi
Coscinodiscus spp.
Amylax triacantha
Amphidinium sphenoides
Protoperidinium granii
Dinophysis rotundata
Glenodinium spp.
Achnanthes taeniata
Ebria tripartita
Actinocyclus octonarius
Flagellates
Melosira arctica
Heterocapsa rotundata
Pennales
Chaetoceros spp.
Chaetoceros wighamii
Gymnodinium sanguineum
Pyramimonas spp.
Dinophysis norvegica
Dinobryon balticum
Dinophysis acuminata
Skeletonema costatum
Aphanizomenon sp.
Wolo/Scrip/Gymn
Peridiniella catenata

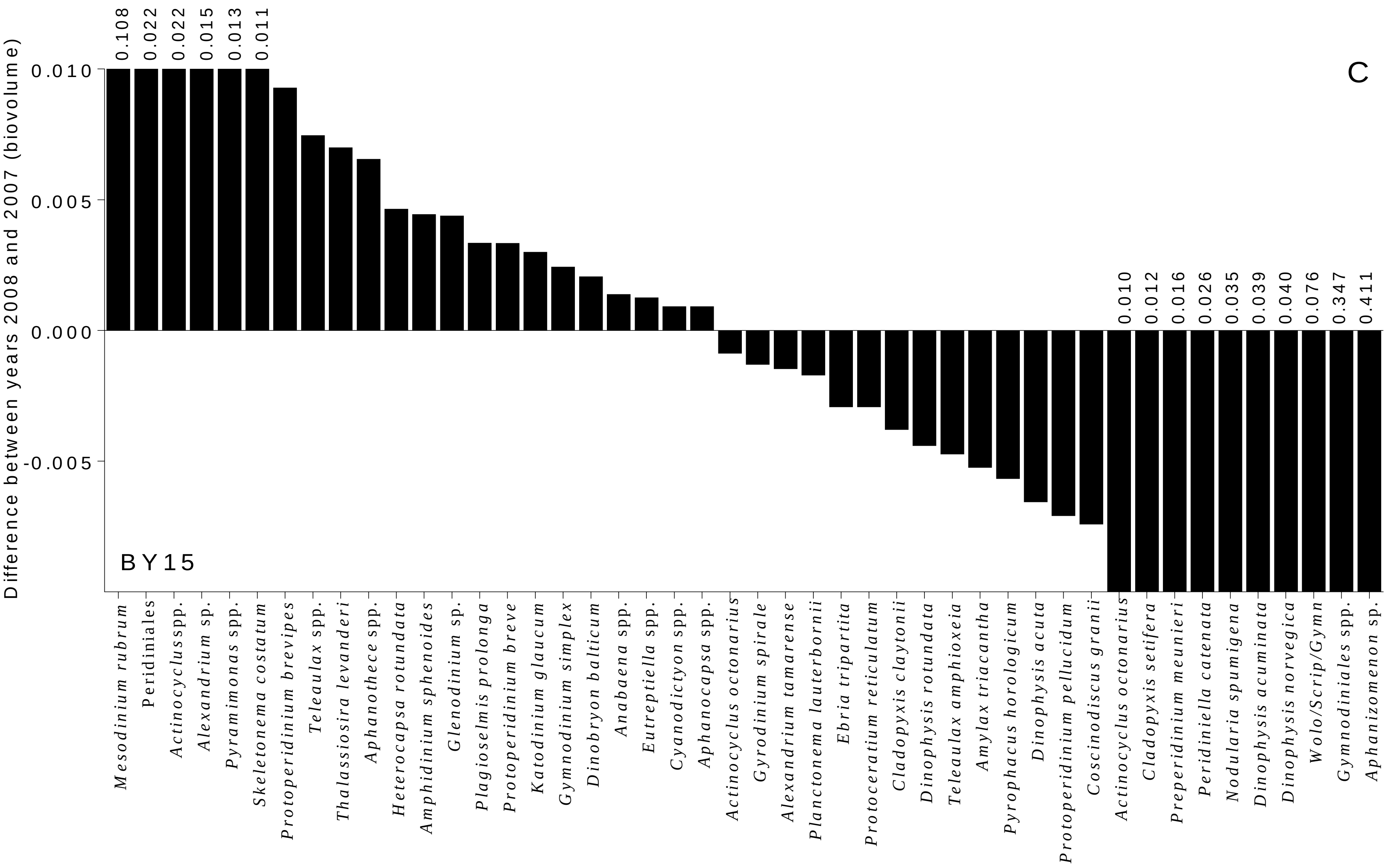
C
BY15
Difference between years 2008 and 2007 (biovolume)
0.010
0.005
0.000
-0.005
0.108
0.022
0.022
0.015
0.013
0.011
0.010
0.012
0.016
0.026
0.035
0.039
0.040
0.076
0.347
0.411
Mesodinium rubrum
Peridiniales
Actinocyclus spp.
Alexandrium sp.
Pyramimonas spp.
Skeletonema costatum
Protoperidinium brevipes
Teleaulax spp.
Thalassiosira levanderi
Aphanothece spp.
Heterocapsa rotundata
Amphidinium sphenoides
Glenodinium sp.
Plagioselmis prolonga
Protoperidinium breve
Katodinium glaucum
Gymnodinium simplex
Dinobryon balticum
Anabaena spp.
Eutreptiella spp.
Cyanodictyon spp.
Aphanocapsa spp.
Actinocyclus octonarius
Gyrodinium spirale
Alexandrium tamarense
Planctonema lauterbornii
Ebria tripartita
Protoceratium reticulatum
Cladopyxis claytonii
Dinophysis rotundata
Teleaulax amphioxeia
Amylax triacantha
Pyrophacus horologicum
Dinophysis acuta
Protoperidinium pellucidum
Coscinodiscus granii
Actinocyclus octonarius
Cladopyxis setifera
Preperidinium meunieri
Peridiniella catenata
Nodularia spumigena
Dinophysis acuminata
Dinophysis norvegica
Wolo/Scrip/Gymn
Gymnodiniales spp.
Aphanizomenon sp.

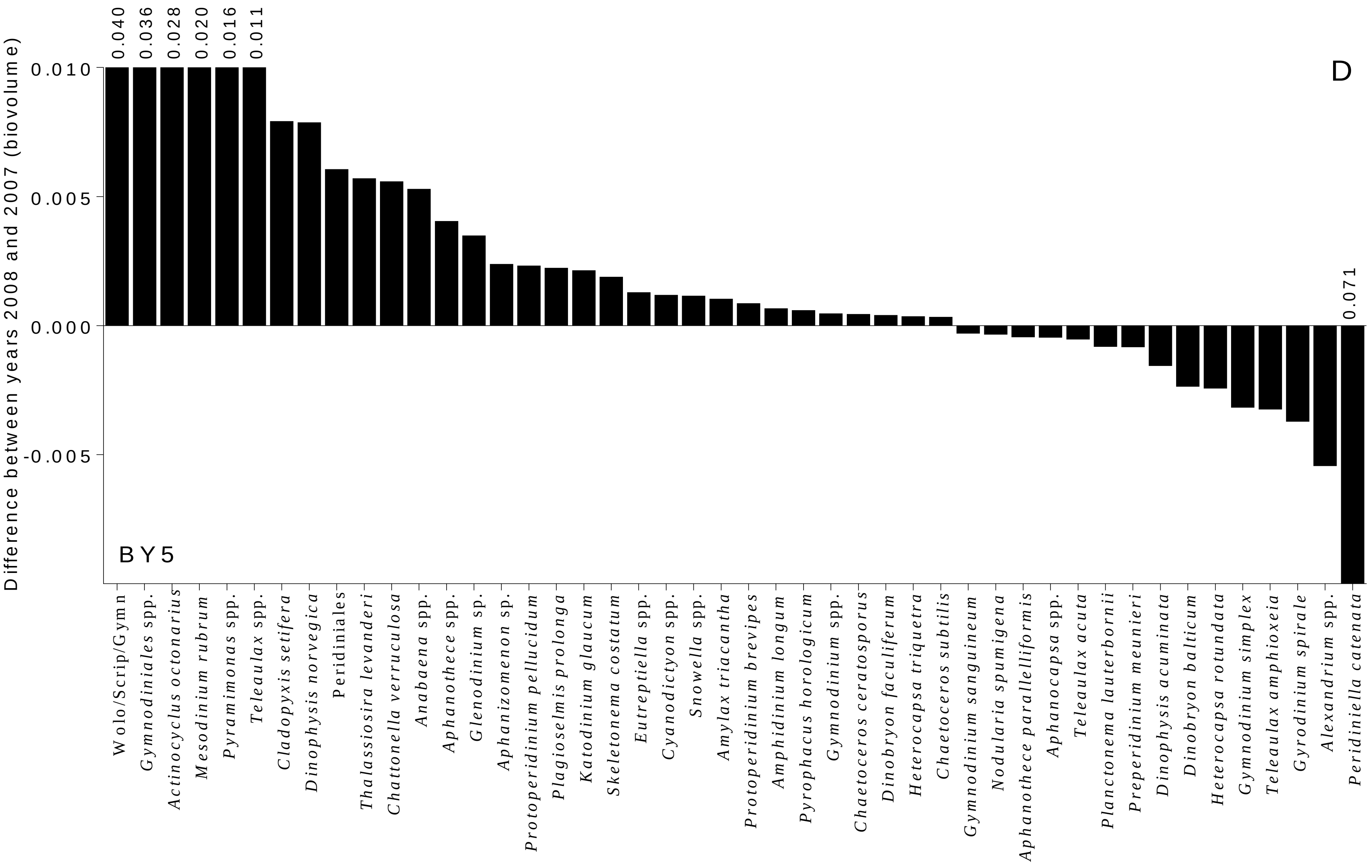

Difference between years 2008 and 2007 (biovolume)
0.010
0.005
0.000
-0.005
0.040
0.036
0.028
0.020
0.016
0.011
0.071
D
BY5
Wolo/Scrip/Gymn
Gymnodiniales spp.
Actinocyclus octonarius
Mesodinium rubrum
Pyramimonas spp.
Teleaulax spp.
Cladopyxis setifera
Dinophysis norvegica
Peridiniales
Thalassiosira levanderi
Chattonella verruculosa
Anabaena spp.
Aphanothece spp.
Glenodinium sp.
Aphanizomenon sp.
Protoperidinium pellucidum
Plagioselmis prolonga
Katodinium glaucum
Skeletonema costatum
Eutreptiella spp.
Cyanodictyon spp.
Snowella spp.
Amylax triacantha
Protoperidinium brevipes
Amphidinium longum
Pyrophacus horologicum
Gymnodinium spp.
Chaetoceros ceratosporus
Dinobryon faculiferum
Heterocapsa triquetra
Chaetoceros subtilis
Gymnodinium sanguineum
Nodularia spumigena
Aphanothece parallelliformis
Aphanocapsa spp.
Teleaulax acuta
Planctonema lauterbornii
Preperidinium meunieri
Dinophysis acuminata
Dinobryon balticum
Heterocapsa rotundata
Gymnodinium simplex
Teleaulax amphioxeia
Gyrodinium spirale
Alexandrium spp.
Peridiniella catenata

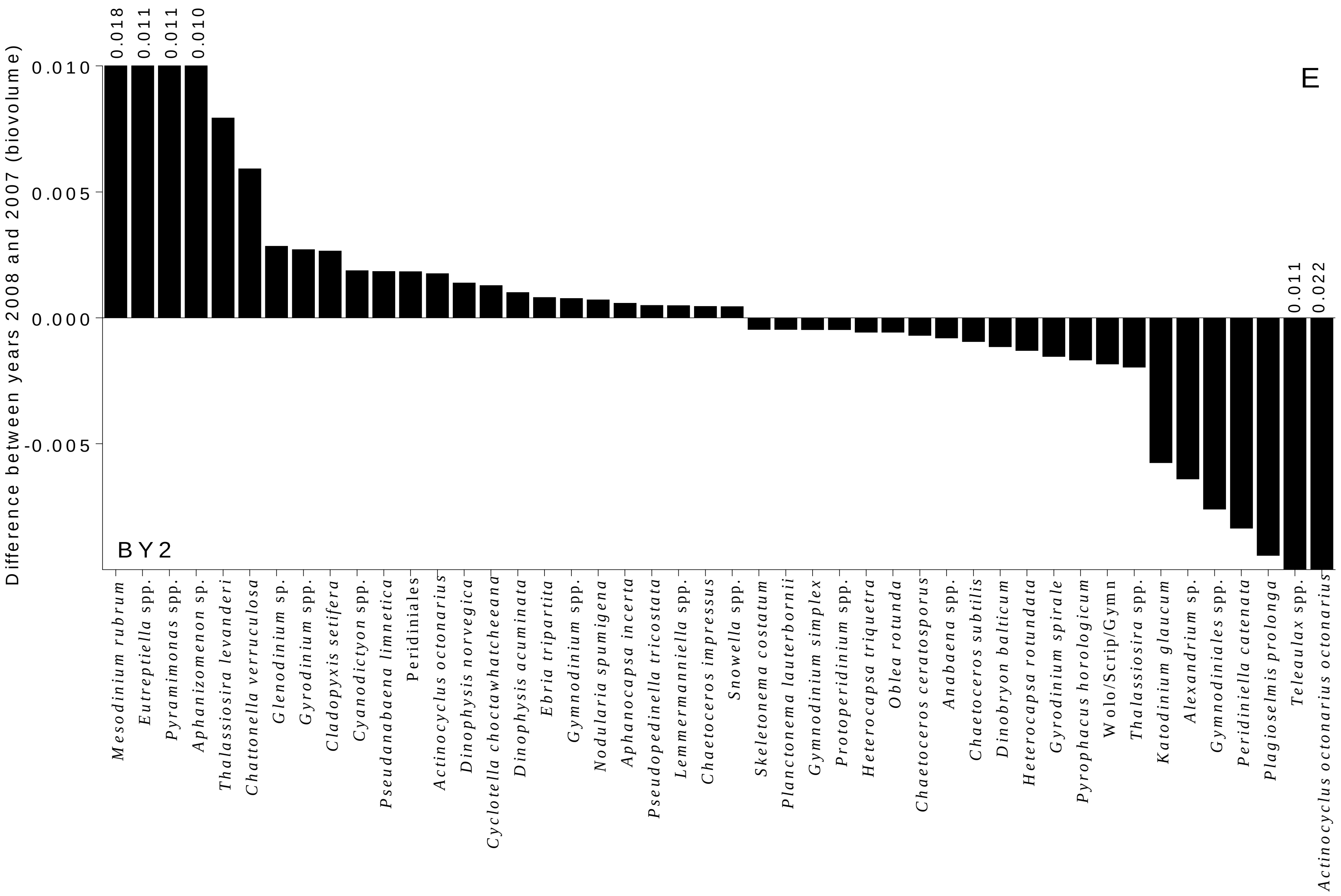
Difference between years 2008 and 2007 (biovolume)
0.010
0.005
0.000
-0.005
0.018
0.011
0.011
0.010
0.011
0.022
E
BY2
Mesodinium rubrum
Eutreptiella spp.
Pyramimonas spp.
Aphanizomenon sp.
Thalassiosira levanderi
Chattonella verruculosa
Glenodinium sp.
Gyrodinium spp.
Cladopyxis setifera
Cyanodictyon spp.
Pseudanabaena limnetica
Peridiniales
Actinocyclus octonarius
Dinophysis norvegica
Cyclotella choctawhatcheeana
Dinophysis acuminata
Ebria tripartita
Gymnodinium spp.
Nodularia spumigena
Aphanocapsa incerta
Pseudopedinella tricostata
Lemmermanniella spp.
Chaetoceros impressus
Snowella spp.
Skeletonema costatum
Planctonema lauterbornii
Gymnodinium simplex
Protoperidinium spp.
Heterocapsa triquetra
Oblea rotunda
Chaetoceros ceratosporus
Anabaena spp.
Chaetoceros subtilis
Dinobryon balticum
Heterocapsa rotundata
Gyrodinium spirale
Pyrophacus horologicum
Wolo/Scrip/Gymn
Thalassiosira spp.
Katodinium glaucum
Alexandrium sp.
Gymnodiniales spp.
Peridiniella catenata
Plagioselmis prolonga
Teleaulax spp.
Actinocyclus octonarius
