# Supplementary material for: Responses of Phyto- and Zooplankton Communities to Prymnesium polylepis (Prymnesiales) Bloom in the Baltic Sea
Source: PLoS One. 2014 Nov 13;9(11):e112985. doi: 10.1371/journal.pone.0112985 (PMC4231118; doi:10.1371/journal.pone.0112985)
Supplement: Figure S3 — Zooplankton taxa showing strong deviations between the study years (2007 vs. 2008) at stns B1 (A), BY31 (B), BY15 (C), BY5 (D), and BY2 (E) in the Baltic proper. Bars show the difference for the entire year (January–December) between year 2008 and year 2007; numbers over the bars at the highest and lowest range indicate values that lie outside the scale on the Y-axis. (PDF) [file pone.0112985.s003.pdf]

Responses of phyto- and zooplankton communities to Prymnesium polylepis (Prymnesiales) bloom in the Baltic Sea

Elena Gorokhova, Susanna Hajdu and Ulf Larsson

Figure S3. Zooplankton taxa showing strong deviations between the study years (2007 vs. 2008) at stns B1 (A), BY31 (B), BY15 (C), BY5 (D) and BY2 (E) in the Baltic proper. Bars show the difference for the entire year (January-December) between year 2008 and year 2007; numbers over the bars at the highest and lowest range indicate values that lie outside the scale on the Y-axis. Note the differences in Y-axis scale among the panels.

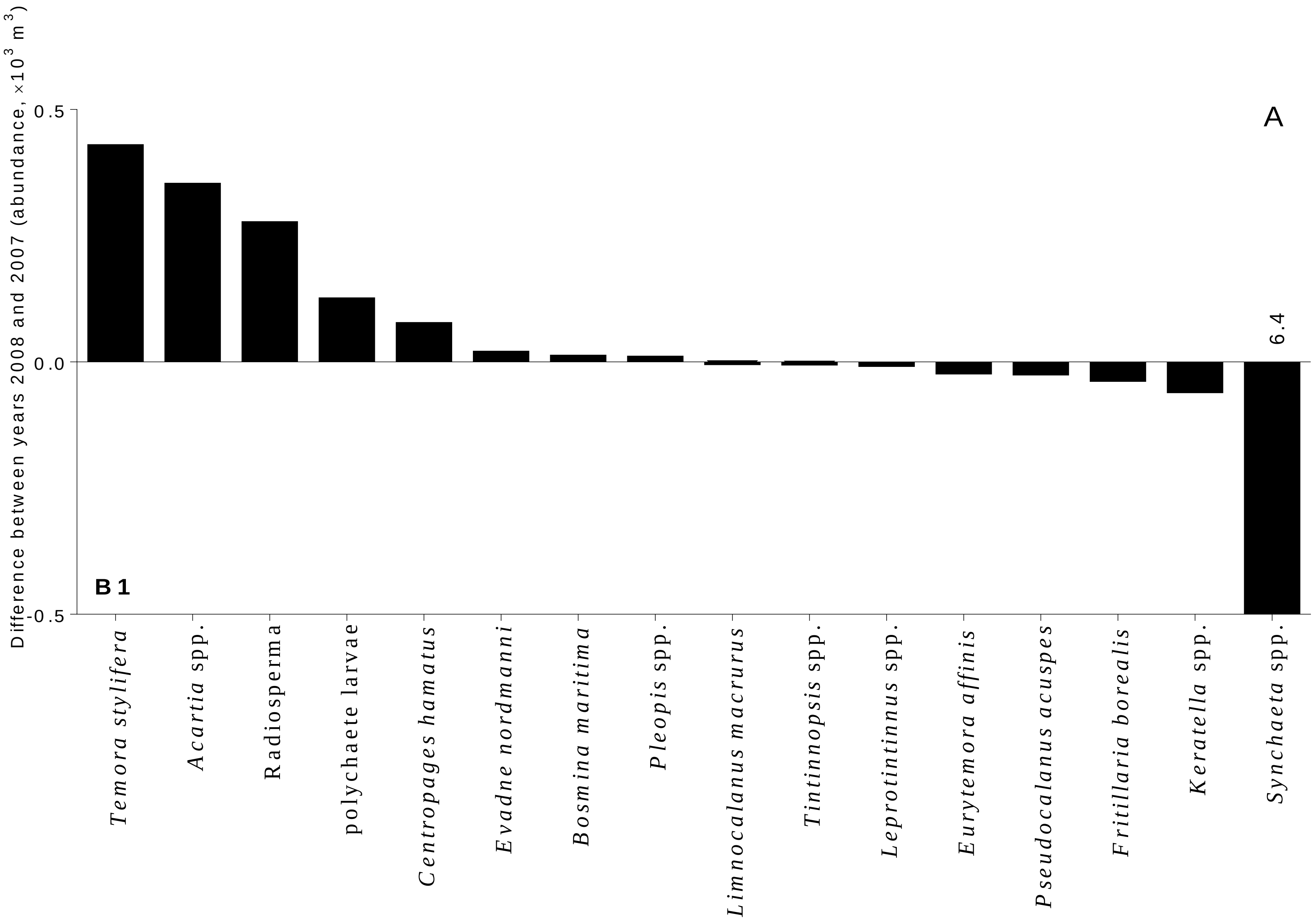

Difference between years 2008 and 2007 (abundance, ×10³ m³)
0.5
0.0
-0.5
A
B 1
6.4
Temora stylifera
Acartia spp.
Radiosperma
polychaete larvae
Centropages hamatus
Evadne nordmanni
Bosmina maritima
Pleopis spp.
Limnocalanus macrurus
Tintinnopsis spp.
Leprotintinnus spp.
Eurytemora affinis
Pseudocalanus acuspes
Fritillaria borealis
Keratella spp.
Synchaeta spp.

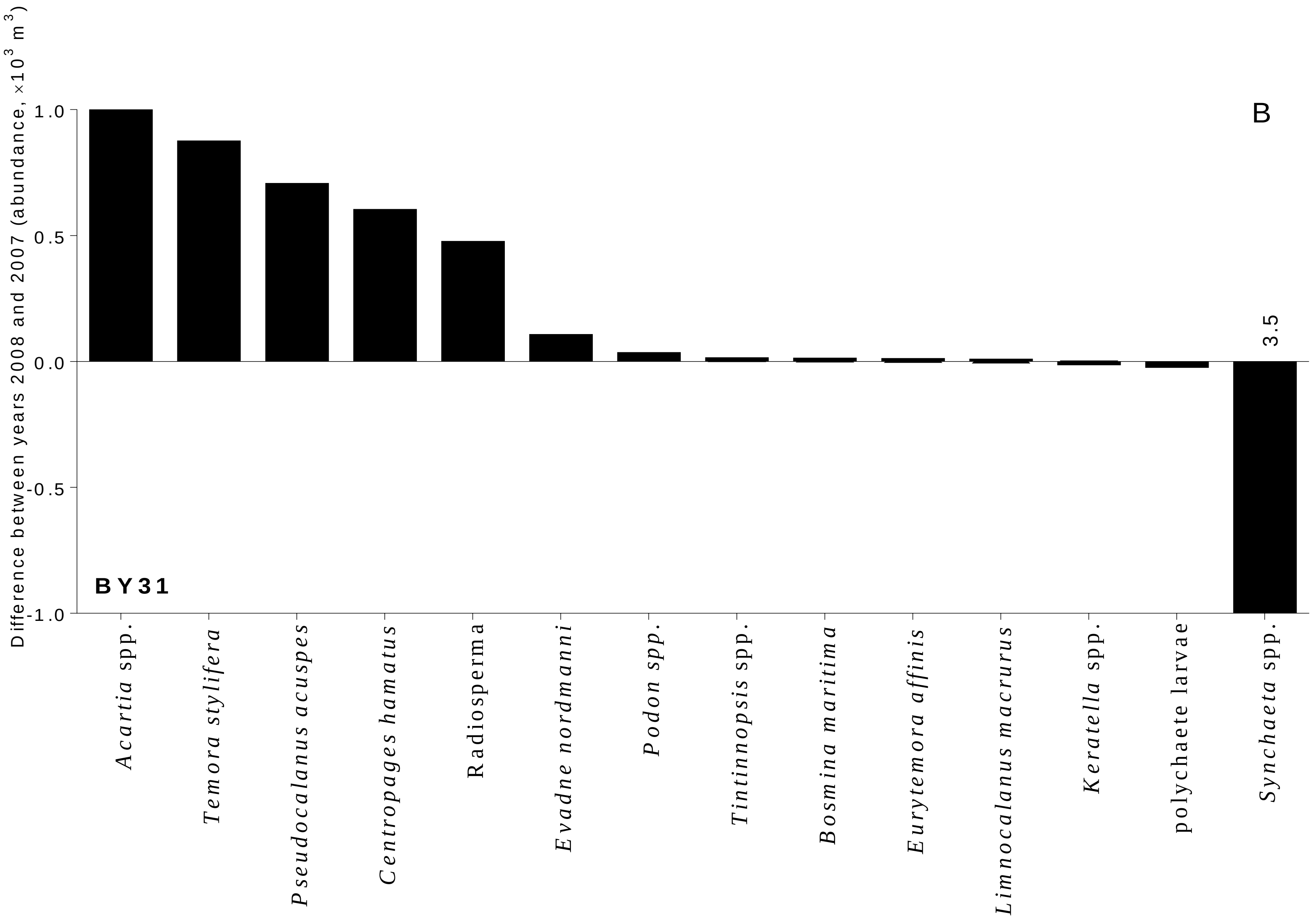
B
BY31
Difference between years 2008 and 2007 (abundance, ×10³ m³)
1.0
0.5
0.0
-0.5
-1.0
3.5
Acartia spp.
Temora stylifera
Pseudocalanus acuspes
Centropages hamatus
Radiosperma
Evadne nordmanni
Podon spp.
Tintinnopsis spp.
Bosmina maritima
Eurytemora affinis
Limnocalanus macrurus
Keratella spp.
polychaete larvae
Synchaeta spp.

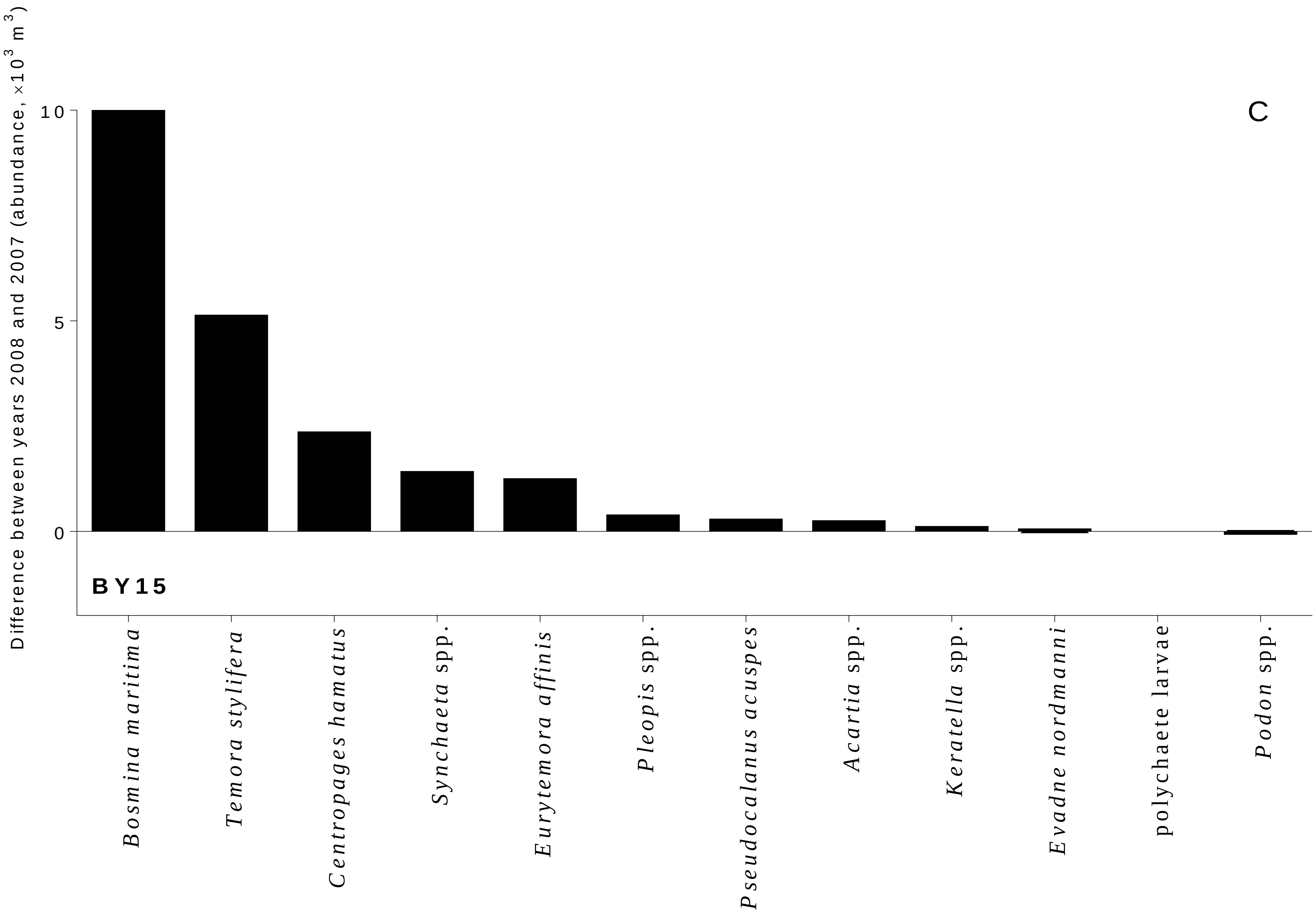

Difference between years 2008 and 2007 (abundance, ×10³ m³)
10
5
0
C
BY15
Bosmina maritima
Temora stylifera
Centropages hamatus
Synchaeta spp.
Eurytemora affinis
Pleopis spp.
Pseudocalanus acuspes
Acartia spp.
Keratella spp.
Evadne nordmanni
polychaete larvae
Podon spp.

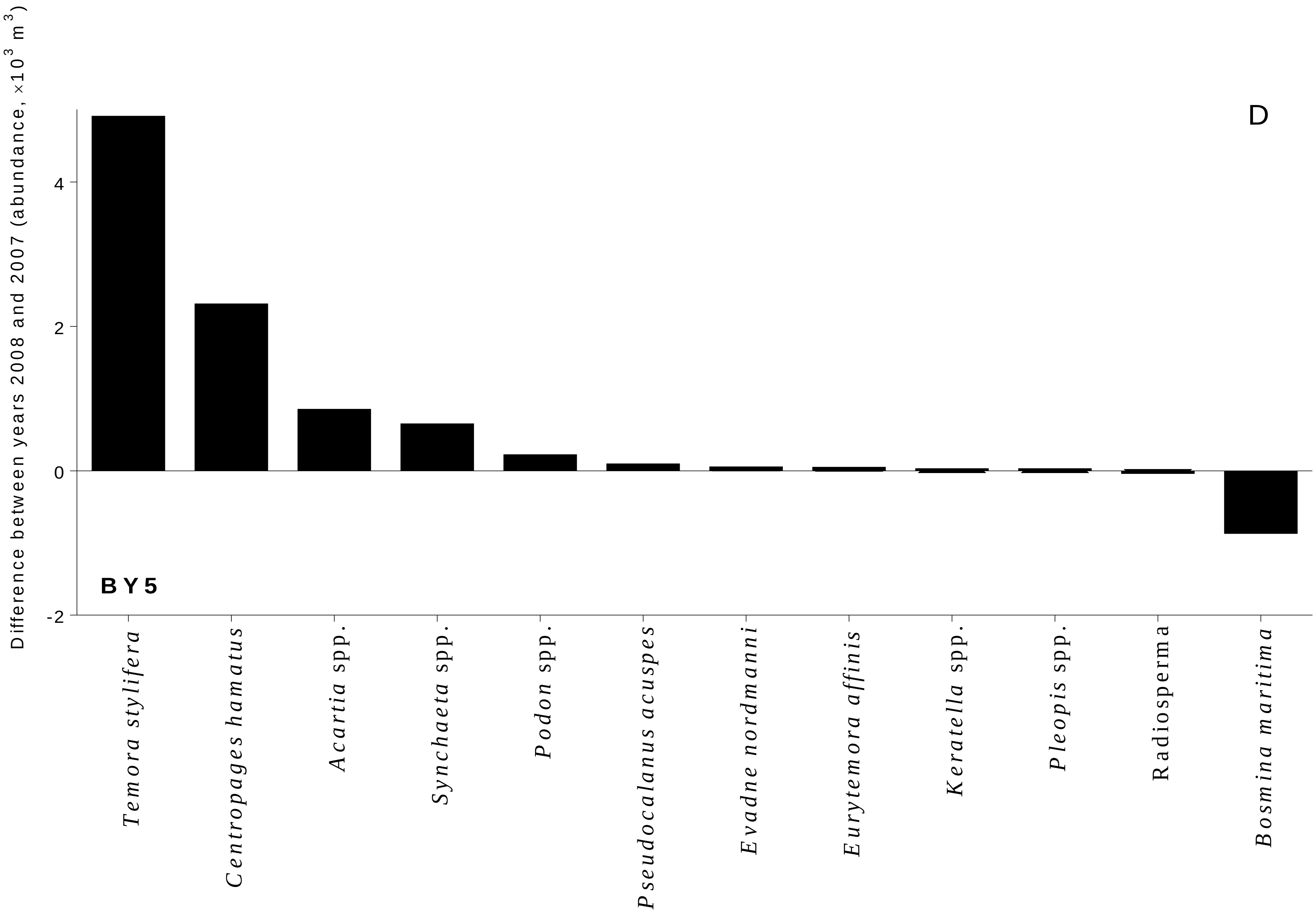

D
BY5
Difference between years 2008 and 2007 (abundance, ×10³ m³)
4
2
0
-2
Temora stylifera
Centropages hamatus
Acartia spp.
Synchaeta spp.
Podon spp.
Pseudocalanus acuspes
Evadne nordmanni
Eurytemora affinis
Keratella spp.
Pleopis spp.
Radiosperma
Bosmina maritima

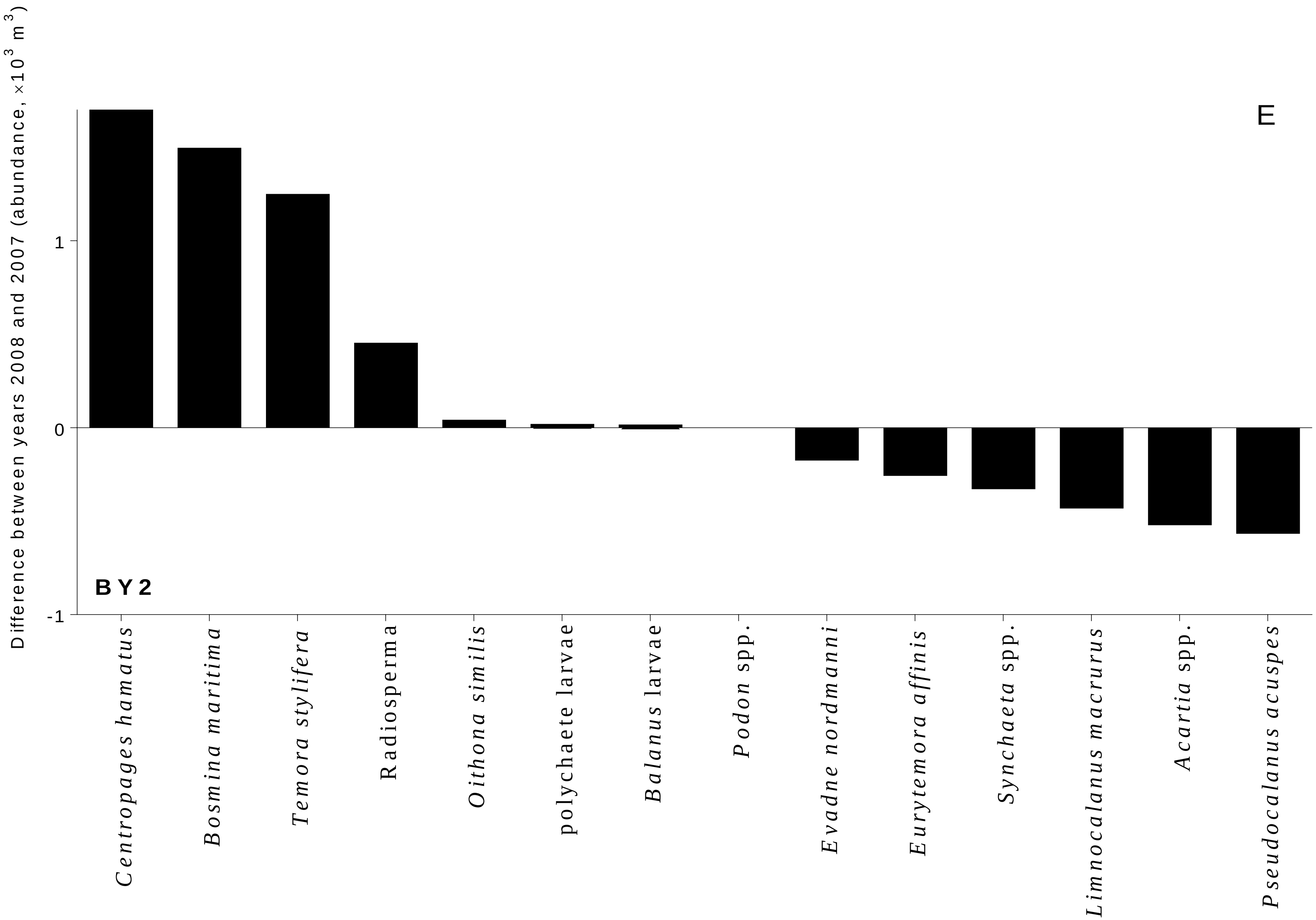
Difference between years 2008 and 2007 (abundance, ×10³ m³)
1
0
-1
E
B Y 2
Centropages hamatus
Bosmina maritima
Temora stylifera
Radiosperma
Oithona similis
polychaete larvae
Balanus larvae
Podon spp.
Evadne nordmanni
Eurytemora affinis
Synchaeta spp.
Limnocalanus macrurus
Acartia spp.
Pseudocalanus acuspes
